# Nationwide Surveillance and Control Measures during Large Measles Outbreak, Bangladesh, 2026

**DOI:** 10.3201/eid3211.260996

**Published:** 2026-11

**Authors:** M. Fazla Rabbi, Pritom Muztahid, Farah Al Mahdi

**Affiliations:** Management Information System, Directorate General of Health Services, Ministry of Health and Family Welfare, Dhaka, Bangladesh (M.F. Rabbi); Institute of Epidemiology, Disease Control and Research, Dhaka (P. Muztahid, F.A. Mahdi)

**Keywords:** Measles, viruses, respiratory infections, outbreak, vaccination, measles-rubella vaccine, epidemiology, surveillance, case-fatality rate, public health emergency, Bangladesh

## Abstract

Nationwide surveillance in Bangladesh during April 2–May 27, 2026, identified a total of 67,079 suspected and 8,834 confirmed measles cases across 8 administrative divisions. Among suspected cases, 472 deaths were reported, and 88 deaths were reported among confirmed cases. Despite high reported vaccine coverage, transmission persisted amid residual immunity gaps. Vaccine stockouts, discontinued vaccination campaigns, and coverage overestimation likely contributed to Bangladesh’s largest recorded measles outbreak.

Measles remains one of the most contagious infectious diseases known, with a basic reproduction number of 12–18, meaning a single case can infect up to 18 susceptible persons in an unvaccinated population (*1*). According to the World Health Organization (WHO), global measles deaths reached ≈95,000 in 2024, occurring mostly among unvaccinated or undervaccinated children <5 years of age (*2*).

Bangladesh has had longstanding immunization success. The Expanded Programme on Immunization was launched in Bangladesh on April 7, 1979, as a pilot program, which included measles vaccine at 9 months of age among its initial 6 childhood vaccines (*3*). Early vaccination coverage was low; <2% through 1984 (*3*). Coverage improved substantially after introducing the measles–rubella (MR) vaccine in 2012 and the second dose at 15 months of age in 2015 (*4*). WHO–United Nations Children’s Fund estimates show that, by 2023, first-dose MR coverage reached 97% and second-dose MR coverage reached 93% (*5*).

On April 4, 2026, Bangladesh notified WHO of a large measles outbreak. The Directorate General of Health Services (DGHS) and Ministry of Health and Family Welfare immediately activated the Health Emergency Operation Center and rapid response teams, who strengthened surveillance, enhanced hospital preparedness, provided vitamin A for suspected and confirmed cases, increased isolation and infection prevention capacity, and fast-tracked vaccine procurement (*6*). We report on the surveillance and control measures and provide recommendations for this ongoing nationwide measles outbreak.

## The Outbreak

In response to the outbreak, the Ministry of Health and Family Welfare followed its established data flow, reporting, and laboratory interoperability plan for measles surveillance (Figure 1). The surveillance system covers facility- to national-level operations for case detection and notification, data and sample collection, reporting, and laboratory testing. 

**Figure 1:**
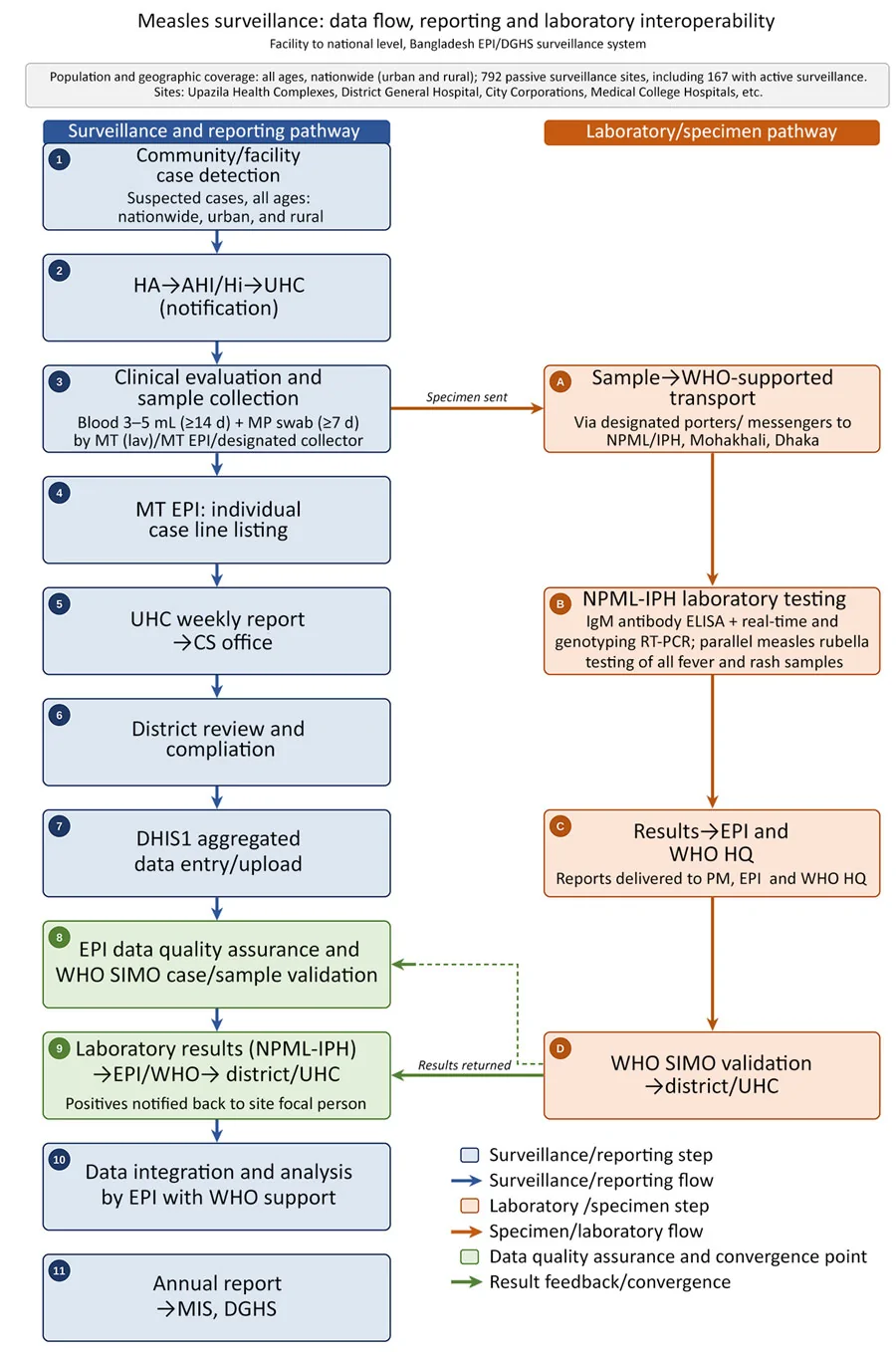
Surveillance, reporting, and laboratory specimen pathways during large measles outbreak, Bangladesh, 2026. AHI, assistant health inspector; DGHS, Directorate General of Health Services; DHIS2, district health information software 2; EPI, Expanded Programme on Immunization; HA, health assistant; HI, health inspector; MIS, Management Information System; MT, medical technologist; NP, nasopharyngeal; NPML-IPH, National Polio, Measles and Rubella Laboratory; SIMO, surveillance and immunization medical officer; UHC, Upazila Health Complex (community health center); WHO, World Health Organization.

### Case Definition

The Bangladesh National Guideline for the Management of Measles case definitions (*7*) were used to identify suspected and confirmed measles cases during the 2026 outbreak. Those guidelines define a suspected case as illness in a person with fever and generalized maculopapular, nonvesicular rash accompanied by cough, coryza, or conjunctivitis or anyone clinicians suspect of having measles (*7*). The guideline defines confirmed cases as illness in persons who test positive for measles, after excluding vaccine-associated symptoms. The National Polio, Measles and Rubella Laboratory confirms cases by using measles-specific IgM ELISA for serum or reverse transcription PCR to detect measles RNA in throat swab, nasopharyngeal swab, or blood samples (*7*).

### Outbreak Scale

Ongoing nationwide active hospital-based surveillance during March 15–May 27, 2026, detected 67,079 suspected and 8,834 confirmed measles cases covering all 64 districts of Bangladesh; 81% of cases were among children <5 years of age. Among the suspected and confirmed cases, >70% (n = 53,463) required hospitalization (*8*). According to the National Guideline for the Management of Measles (*7*), only patients with complications or those with severe or complicated measles cases were hospitalized. Nonetheless, the number of hospitalized patients posed a substantial strain on hospital facilities during the peak transmission weeks. A total of 472 deaths (case-fatality rate [CFR] 0.88%) among suspected measles cases and 88 deaths (CFR 0.99%) among confirmed cases were reported during March 15–May 27, 2026 (*8*). 

### Epidemic Trajectory

The daily epidemic curve from April 2–May 27, 2026, showed a peak of 1,829 cases reported in a single day on April 9, 2026 (Figure 2) (*9*). Reported cases reflected early explosive spread before initiation of the nationwide vaccination response on April 5, 2026. Using case counts from the DGHS and 2022 Bangladesh census population data (*10*), we calculated incidence rates for each division. Dhaka division reported the highest incidence (82.80 cases/100,000 population), followed by Barishal (65.63/100,000) and Rajshahi divisions (35.22/100,000); Rangpur reported the lowest incidence (7.63/100,000) (Figure 3).

**Figure 2:**
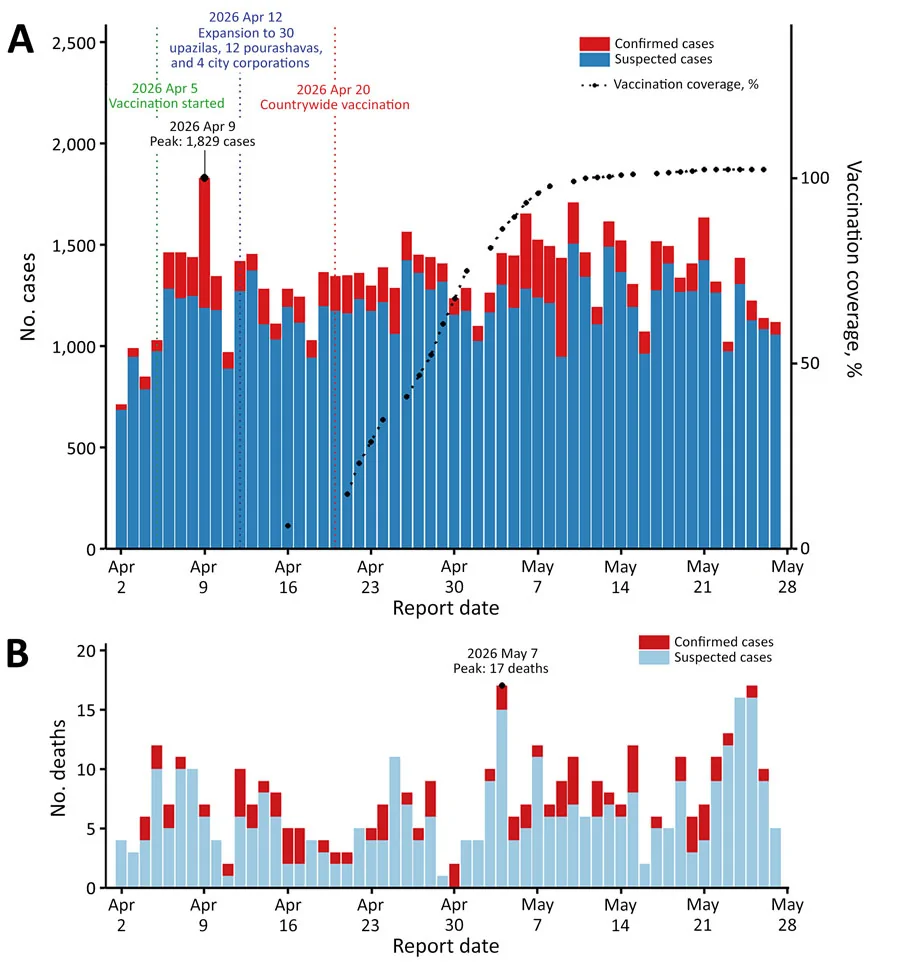
Daily epidemic curve showing the distribution of suspected and confirmed cases during large measles outbreak, Bangladesh, 2026. A) Overall confirmed and suspected cases during April 2–May 27, 2026, overlaid with coverage during vaccination campaign. Vaccination coverage shows >100% because Bangladesh targeted children 6–59 months of age for MR vaccination, regardless of vaccination status to achieve measles control goals. B) Number of deaths among suspected and confirmed cases. Reported cases peaked on April 9, 2026, with 1,829 cases reported in a single day, reflecting early explosive spread before expansion to a nationwide vaccination response on April 20, 2026.

**Figure 3:**
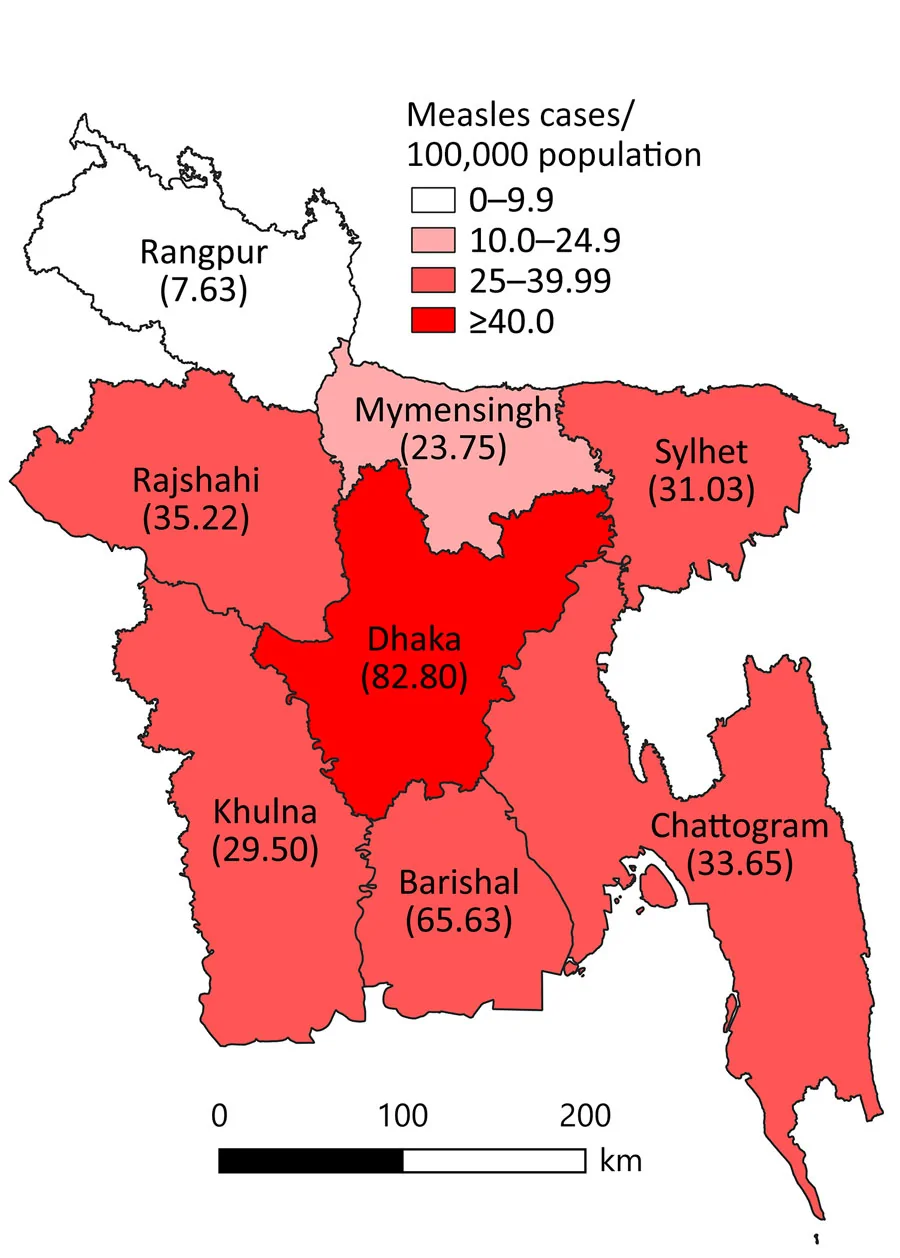
Geographic distribution during large measles outbreak, Bangladesh, 2026. Map shows cases/100,000 population for each division during March 15–May 27, 2026; the outbreak is ongoing. Data were sourced from Bangladesh Directorate General of Health Services and Bureau of Statistics (*8*,*9*,*12*,*13*) Population and Housing Census 2022 (*10*).

### Vaccination Campaign Response

According to WHO, supplementary immunization campaigns deliver vaccination to all targeted persons, regardless of their vaccination status, to rapidly raise population-level immunity and reduce the number of susceptible persons (*11*). To achieve measles control goals, Bangladesh targeted children 6–59 months of age for MR vaccination.

The Bangladesh National Immunization Technical Advisory Group approved targeted vaccination March 30, 2026, and the targeted MR catch-up campaign commenced in 30 upazilas (subdistricts) of 18 priority districts on April 5, 2026. By April 12, coverage expanded to 30 upazilas, 1 pourashava (municipality), and 4 city corporations. A nationwide MR vaccination campaign was launched on April 20, 2026, and targeted ≈18,015,064 children 6–59 months of age (*6*).

Initial DGHS reports from April 16, 2026, showed a 6% coverage rate at the start of the campaign. Rapid momentum over the subsequent 6 weeks culminated on May 13, 2026, when the campaign officially surpassed 100% of the targeted baseline coverage (*12*,*13*) (Figure 2, panel A).

## Conclusions

Bangladesh is experiencing its largest measles outbreak on record, and a total of 75,913 cases and 560 deaths were recorded in 56 days (*8*). The ongoing outbreak shows no sign of slowing, and the highest cumulative weekly death toll came during May 21–27, 2026, the last week recorded before this report. This soaring disease burden signifies several observations.

First, the highest weekly case count (10,427 cases with 62 deaths) was recorded May 7–13 (https://doi.org/10.17605/OSF.IO/RHQUS), when vaccination coverage exceeded 100% of the administrative target (Figure 2). That pattern demonstrates a large preexisting susceptible population. The impact of emergency mass vaccination typically becomes evident within 2–4 weeks as vaccinated children acquire protection and the susceptible pool diminishes. The practical lesson is that herd protection thresholds must be maintained continuously through routine and catch-up vaccination, not reactive campaigns.

Second, the large gap between suspected and laboratory-confirmed cases (≈7:1) partly reflected the usual surveillance practice, since not every suspected case required laboratory confirmation. However, that gap might also reflect limited testing capacity because only 1 laboratory facility serves the country. Moreover, a case-level linkage between tested specimens and surveillance records was not available, so the positive predictive value could not be calculated. The reported CFR in Bangladesh during this outbreak was 0.88% for suspected cases and 0.99% for confirmed cases, which should be interpreted cautiously given that limitation. Those rates were higher than rates reported in the United States and Europe (0.13% each) but within the 1.5%–5.35% range reported in some low- and lower middle–income settings (*14*–*16*). However, the observed CFR of Bangladesh reflected cases reaching healthcare facilities rather than the true population-level rate. Consequently, limited community-based reporting of mild illness likely biased the CFR upward.

Third, the high (70%) hospitalization rate among reported cases should be discussed cautiously; measles cases reported through the surveillance systems typically represent a fraction of the true number of cases because many children are not assessed by clinicians (*17*). In addition, when medical care is sought, measles might be misdiagnosed or cases might not be reported to authorities (*17*).

Fourth, measles cases were reported across all 8 divisions from the beginning of the outbreak, indicating widespread transmission amid nationwide immunity gaps. Contributing factors include the 2024–2025 MR vaccine stockout, gaps in routine immunization, and the absence of nationwide MR campaigns since 2020 (*6*), a pattern also seen in other low- and middle-income countries after immunization disruptions (*18*,*19*). Part of that gap might be further underestimated by reporting discrepancies: Bangladesh’s administrative coverage, the reported number of vaccine doses administered divided by the estimated target population, still calculated on the basis of 2011 census denominators, has exceeded 100% in places, pointing to denominator underestimation and overreporting that likely mask the true extent of population immunity gaps (*20*).

In line with WHO guidance (*21*), we recommended several measures to control the outbreak. Those measures included continuing the MR campaign in periurban and other low-coverage areas to reach underimmunized populations; strengthening community engagement and caregiver education to increase vaccine demand and address misinformation; ensuring routine Expanded Programme on Immunization service delivery with adequate supply, with special attention to low-coverage districts; and strengthening real-time sentinel measles surveillance to detect future outbreaks early.
